# Detection of hospital environmental contamination during SARS-CoV-2 Omicron predominance using a highly sensitive air sampling device

**DOI:** 10.3389/fpubh.2022.1067575

**Published:** 2023-01-10

**Authors:** Kai Sen Tan, Alicia Xin Yu Ang, Douglas Jie Wen Tay, Jyoti Somani, Alexander Jet Yue Ng, Li Lee Peng, Justin Jang Hann Chu, Paul Anantharajah Tambyah, David Michael Allen

**Affiliations:** ^1^Biosafety Level 3 Core Facility, Yong Loo Lin School of Medicine, National University of Singapore, Singapore, Singapore; ^2^Department of Microbiology and Immunology, Yong Loo Lin School of Medicine, National University of Singapore, Singapore, Singapore; ^3^Infectious Disease Translational Research Programme, Yong Loo Lin School of Medicine, National University of Singapore, Singapore, Singapore; ^4^Department of Otolaryngology, Yong Loo Lin School of Medicine, National University of Singapore, Singapore, Singapore; ^5^Department of Medicine, Division of Infectious Diseases, National University Hospital, Singapore, Singapore; ^6^Department of Emergency Medicine, National University Hospital, Singapore, Singapore; ^7^Collaborative and Translation Unit for Hand, Foot and Mouth Disease (HFMD), Institute of Molecular and Cell Biology, Agency for Science, Technology and Research, Singapore, Singapore

**Keywords:** air-sampling, SARS-CoV-2, Omicron, surveillance, mass screening

## Abstract

**Background and objectives:**

The high transmissibility of SARS-CoV-2 has exposed weaknesses in our infection control and detection measures, particularly in healthcare settings. Aerial sampling has evolved from passive impact filters to active sampling using negative pressure to expose culture substrate for virus detection. We evaluated the effectiveness of an active air sampling device as a potential surveillance system in detecting hospital pathogens, for augmenting containment measures to prevent nosocomial transmission, using SARS-CoV-2 as a surrogate.

**Methods:**

We conducted air sampling in a hospital environment using the AerosolSense^TM^ air sampling device and compared it with surface swabs for their capacity to detect SARS-CoV-2.

**Results:**

When combined with RT-qPCR detection, we found the device provided consistent SARS-CoV-2 detection, compared to surface sampling, in as little as 2 h of sampling time. The device also showed that it can identify minute quantities of SARS-CoV-2 in designated “clean areas” and through a N95 mask, indicating good surveillance capacity and sensitivity of the device in hospital settings.

**Conclusion:**

Active air sampling was shown to be a sensitive surveillance system in healthcare settings. Findings from this study can also be applied in an organism agnostic manner for surveillance in the hospital, improving our ability to contain and prevent nosocomial outbreaks.

## Introduction

Severe Acute Respiratory Syndrome Coronavirus 2 (SARS-CoV-2), which causes Coronavirus Disease 2019 (COVID-19), has profoundly disrupted life globally since December 2019 (1). Implementation of public health control measures since the emergence of SARS-CoV-2, including mass testing, contact tracing, border controls, quarantine, safe distancing, enhanced cleaning, and mass vaccination, while important, could not achieve sustained elimination of SARS-CoV-2 (2–4). In addition, the potential airborne transmission of SARS-CoV-2 has further hampered efforts to stem its circulation worldwide (5, 6). In response, most regions have transitioned away from a zero COVID-19 strategy toward endemicity by opening borders and relaxing social distancing measures. However, frequent testing to identify infected individuals remains a cornerstone in curbing community transmission and has been modeled to be an effective intervention for countries transitioning toward endemicity (7). This is especially important in hospital settings where nosocomial SARS-CoV-2 and other infectious pathogens continue to pose a risk to vulnerable populations and healthcare workers (8–10). Therefore, an effective hospital surveillance programme, which allows rapid implementation of appropriate containment measures and re-allocation of healthcare resources is vital to mitigate preventable loss of life and the burden on the healthcare sector (7, 10).

SARS-CoV-2 surveillance began with individual-based testing early in the pandemic, using reverse transcription real-time polymerase chain reaction (RT-qPCR) to detect viral RNA from nasopharyngeal swabs samples (11, 12). Other screening methods have also been employed, such as thermography, antigen rapid tests (ART), and volatile organic compound detection (12). Subsequently, mass surveillance efforts, such as sampling wastewater for the presence of SARS-CoV-2 nucleic acid, have been used to complement individual testing efforts (13). Wastewater surveillance has historical precedence and current relevance in poliomyelitis surveillance and has been highly effective in identifying infection clusters or emergence of COVID-19 globally (14–17). However, in endemic settings where community caseloads are low, individual testing is resource intensive yet of limited yield. Wastewater surveillance involves coverage of large areas that may be non-specific to the target location and cannot inform immediate infection containment measures due to the retrospective nature of the surveillance. Hence, neither surveillance methods may be suitable in monitoring nosocomial airborne pathogens such as SARS-CoV-2. Therefore, we and others have explored air sampling as a mass surveillance system that can be conducted near real-time in communal settings, especially for healthcare environments (18–20).

The COVID-19 pandemic has exposed a gap in hospital surveillance where there is a lack of a low cost and easily deployable system for the rapid detection of nosocomial pathogens (including SARS-CoV-2) in non-pandemic times. While individual testing surveillance is effective, it is proving to be unsustainable (21, 22). Additionally, an effective mass sampling system would allow more rationale PPE usage with its positive impact on healthcare worker's experience and the volume of biohazard waste (23). Studies on SARS-CoV-2 have shown that traces of the virus shed from infected individuals, both symptomatic or asymptomatic, can be detected in aerosols and on surfaces (18, 24, 25). In this regard, air sampling may be suitable as a mass surveillance tool to detect airborne pathogens in the environment to indicate the presence of infected individuals in specific locations. An advantage of air surveillance is that it bypasses test-seeking behavior and could detect pathogens from multiple individuals as a pooled source. In addition, air samplers can be rapidly deployed with short time-to-results to inform containment measures or as a near real-time surveillance system.

Therefore, with the high SARS-CoV-2 case load fueled by the high transmissibility of the Omicron variant of concern (VOC) and its subvariants (26, 27), we tested a high flowrate aerial sampler (AerosolSense^TM^) as a surveillance tool in a healthcare facility (National University Hospital) in Singapore housing patients with and without COVID-19. We aimed to demonstrate a proof-of-concept, using detection of the highly transmissible Omicron VOC as a surrogate, to elucidate the feasibility of aerial sampling for pathogen surveillance in healthcare settings. Our findings may be applied for mass surveillance of other airborne pathogens to augment monitoring in the hospital as a preventative measure for nosocomial infections and outbreaks.

## Materials and methods

The study was conducted from January to March 2022 when Singapore's first Omicron wave occurred, with Omicron BA.1 and then BA.2 representing almost all isolates by February, according to the SARS-CoV-2 pathogen tracking in Nextstrain database (28). We sampled the air and surface environments of facilities that exclusively housed patients positive for SARS-CoV-2 by RT-qPCR (C+ facilities) to compare both sampling methods. We also sampled the air environment of a facility not known to house COVID-19 patients (C- facilities) for further comparison of aerial sampling sensitivity. All sampling was performed in the National University Hospital (NUH), an academic quaternary medical center in Singapore. As there was no direct use of patient samples or identifiers involved, the study was given a waiver of approval by the Domain Specific Review Board (DSRB) of the National Healthcare Group (NHG).

### Description of sampling locations

Air sampling was conducted in three different C+ facilities: the negative pressure (NP) isolation wards, open-cohort wards, and the emergency department annex (EDA). We sampled both patient areas and staff areas (designated clean areas separated from the patient areas) in all three C+ facilities. All patients in C+ facilities were admitted after a positive COVID-19 test by RT-qPCR (with cycle threshold, Ct of < 25). Both adults and children were admitted to these three facilities. All C+ facilities within NUH were at capacity with COVID-19 patients at the time of sampling. Disease severity of the patients occupying the wards during the study ranged from asymptomatic to moderate to severe infection requiring oxygen. We did not sample the intensive care units where severe cases including those on mechanical ventilation were housed.

The NP isolation ward consists of 21 single occupancy negative pressure rooms, each with 14 air changes per hour (ACH) of fresh air with no recirculation. Each room has a dedicated toilet, and four rooms have anterooms (designated as clean areas with no PPE requirement). The room temperature and relative humidity were maintained at 23°C and 60%, respectively, while in use. We only sampled rooms housing RT-qPCR confirmed COVID-19 patients.

The open-cohort ward had been re-purposed to exclusively care for COVID-19 patients. The entirety of the patient ward is designated as “contaminated” and is entered *via* an electronically activated sealed door. Healthcare staff must don full PPE consisting of N95 masks, long-sleeved gowns, gloves, and goggles once they enter the ward. All patients wore regular surgical masks whenever possible in the open-cohort ward. The open-cohort ward consists of seven open cubicles, each housing six patients, with one toilet and shower per cubicle. There are two single rooms in the ward with their own bathrooms. A common corridor connects the cubicles and single rooms. The ward employs natural ventilation with open windows and ceiling fans. The temperature and relative humidity fluctuate according to the outdoor air of this tropical climate with a range of 24–30°C and 60–90%, respectively. Non-patient care areas are designated clean staff areas according to hospital guidelines, which are located immediately adjacent to the wards but are outside of the patient areas. Staff do not don full PPE in staff areas but don medical-grade surgical masks as per hospital guidelines.

The EDA was built toward the end of 2021 to accommodate the surge in patients during the Omicron wave. Patients in the EDA were either suspected or proven COVID-19 patients and remained in the EDA while awaiting SARS-CoV-2 swab RT-qPCR or ART results to triage for transfer to community COVID-19 facilities, admission to hospital or discharged home. The EDA contains 19 cubicles, each with floor to ceiling sliding doors and a single exhaust vent per room which exhausts HEPA filtered air to the exterior. The corridors maintain an overall positive pressure relative to the individual rooms. A healthcare staff workstation is located between two central corridors in the middle of the EDA. A staff rest area with dedicated ingress and egress is separated from the rest of EDA by a door. The staff rest area has dedicated ceiling exhaust and maintains an overall positive pressure relative to the rest of the EDA. Portable HEPA filter air purifiers were also placed in the staff rest area. ACH in the EDA is 12 ACH (2 fresh, 10 filtered and recycled). Both the staff workstation and rest area are designated clean areas where staff do not don full PPE but have N95 mask on as per hospital guidelines. During the sampling period, the EDA was confirmed to house RT-qPCR confirmed COVID-19 patients and only rooms housing these patients were sampled, in addition to the staff areas.

The C- facility is an open-cohort ward that did not house known COVID-19 patients. In the C- facilities, all patients wore surgical masks whenever possible, while staff don N95 masks, as per prevailing national guidelines in Singapore. Staff do not don full PPE. To ensure that the C- facilities have no overt transmission, all patients were tested on admission, while staff who were symptomatic or identified as a close contact were tested to ensure that they are negative for SARS-CoV-2 *via* RT-qPCR or ART. Visitors were not allowed during the period of sampling. This remained the standard practice in hospitals in Singapore throughout the sampling period. The negative controls were obtained from 2 rooms located within an adjacent office building for staff that are separate from the hospital, as non-hospital location negative controls.

All sampling areas, including the C- facilities, were under restricted access limited to patients and healthcare workers caring for these patients. All staff were required to perform twice-weekly self-testing ART. No untested patients or healthcare workers entered these restricted areas during sampling periods, as part of Singapore's COVID-19 restriction guidelines for safeguarding healthcare personnel. Due to the restricted access of only necessary healthcare workers that are present in all wards, traffic through the sampling locations was hence comparable.

**Figure 1:**
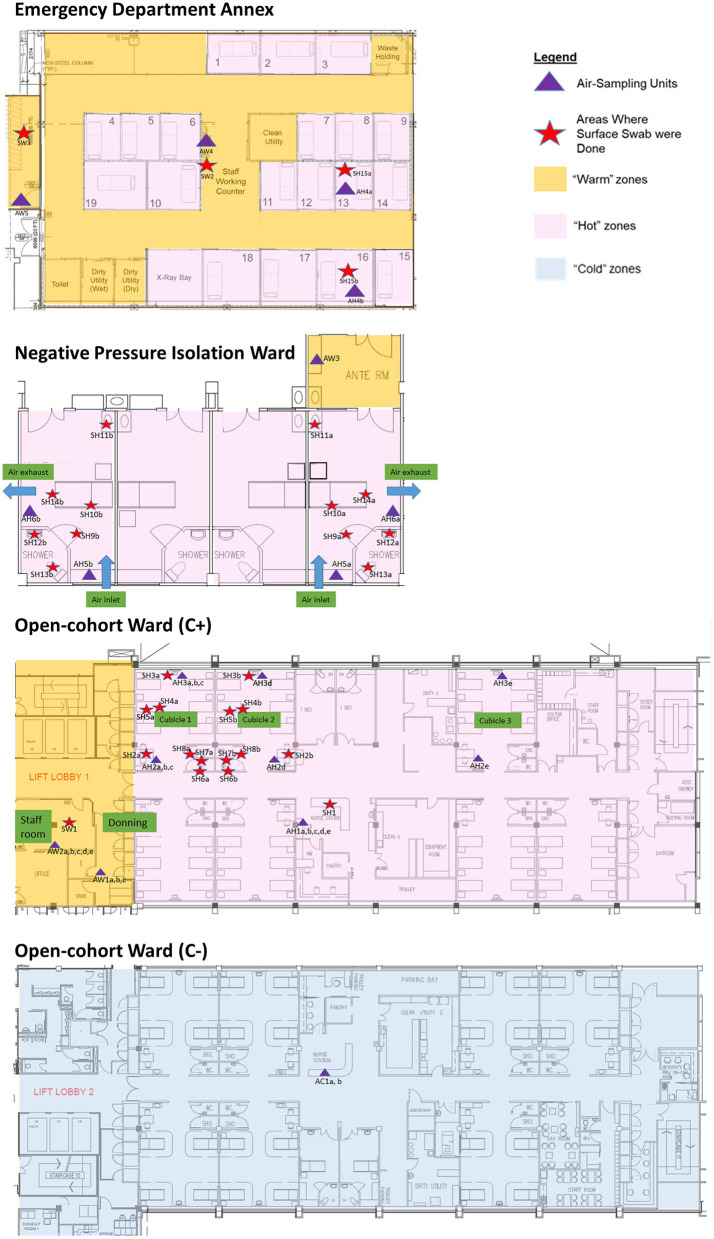
Sampling plans (air and surface) and location in Hot, Warm, and Cold areas of the emergency department annex, negative pressure isolation ward, and open-cohort wards (C+ and C-).

The sampling locations were categorized based on C+ patient traffic for analysis of detection sensitivity. Hot areas were designated “dirty” areas based on active C+ patient movements, where staff entering the areas don full PPE. Warm areas were designated “clean” areas with no C+ patient movements, where there are no full PPE requirements for staff other than N95 mask, but are areas directly adjacent to Hot areas. Warm areas include the anterooms, staff workstations and rest areas under positive pressure. Cold areas were designated “clean” areas not housing C+ patients, have no full PPE requirements, and are located apart from the Hot areas. Negative controls were taken from an adjacent building as a non-hospital location control. The schematics of the C+ and C- facilities are shown in Figure 1.

### Air sampling

All air sampling was conducted with a cartridge-based AerosolSense^TM^ air sampler (Thermo Fisher Scientific) at a rate of 200 L/min through a vertical collection pipe and impacted onto the collection cartridge. The AerosolSense^TM^ is designed to collect aerosolized particles with a diameter between 0.1 and 15 μm. The air sampler has been used in a variety of environmental settings to detect and quantify SARS-CoV-2 RNA. A single-use sampling cartridge containing 2.5 cm collection substrates in a sponge was installed into the sampler. Air samples were collected from all C+, C- and negative control areas during an overnight 14-h period (6 pm−8 am the next day), or during a 2-h period (7 am−9 am). The exception would be air samples from EDA, which were collected over 24 h (8 am−8 am the next day). Sampling times and device placements were selected to minimize disruption to clinical service and ensure minimal accidental contamination or movement of the samplers. In the NP isolation ward, air samplers were placed in the patient's room, < 2.5 m away from the patient (Hot samples), and in the anteroom (Warm samples). In the EDA, air samplers were placed inside patient rooms, < 2.5 m away from the patient (Hot samples) and two staff areas (Warm samples). In the C+ open-cohort ward, air samplers were placed within patient cubicles < 2 m away from the nearest patient (Hot samples), common areas < 10 m away from the nearest patient (Hot samples), and staff areas separated by one or more doors from patient areas, located < 14 m away from the nearest patient (Warm samples). Two air samplers were placed in the C- open-cohort ward for 2 and 14 h each (Cold samples). Two air samplers were placed in office rooms not connected to the hospital (negative control samples). A total of 36 air samples were collected: 23 in C+ open-cohort ward, 5 in NP isolation rooms, 4 in EDA, 2 in C- open-cohort ward, and 2 negative controls. In addition, separate testing was done using N95 respirators (*n* = 1 Hot; *n* = 1 Warm) and surgical masks (*n* = 1 Hot; *n* = 1 Warm) covering the inlet of the sampler to simulate mask wearing on the effect of detection (Supplementary Figure 1).

### Surface sampling

Surface samples were obtained using sterile swabs moistened with viral Universal Transport Media (UTM; Noble Biosciences). Each surface sample was collected by swabbing a 10 × 10 cm site. Swab samples were collected at the completion of the air sampling cycle in each of the three C+ facilities (Hot and Warm samples only) and collected at similar locations to where air sampling was conducted, for comparison. The chosen swab sites had not been cleaned for at least 8 h prior to swabbing. Surfaces of both patient care areas, including toilets (Hot samples), and staff areas (Warm samples) were swabbed. Surface sampling were not conducted in C- facilities for Cold samples collection based on previous data where there was absence of detection in C- areas (18). In addition, the Cold sample collection was conducted mainly to assess the detection sensitivity of air sampling. All surface swab samples were stored in 1.8 ml of viral UTM. A total of 32 surface samples were collected: 12 in NP isolation rooms, 16 in C+ open-cohort ward, and 4 in EDA. All sampling processes and workflow are summarized in Supplementary Figure 2.

### Sample processing

All samples were processed on the same day of collection in the National University of Singapore biosafety level 3 laboratory. For air samples, the collection sponge was removed from the cartridge and placed in 800 μl of Dulbecco's Modified Eagle Medium (Gibco), vortexed, and aliquoted into screw-cap tubes. For surface swabs, the UTM tubes were vortexed and aliquoted into screw-cap tubes. All samples were stored at −80°C until analysis.

### Quantification of viral RNA

RNA was extracted using the QIAamp MinElute Virus Spin Kit (Qiagen) according to manufacturer's instructions, eluted in 50 μl of nuclease-free water, and stored at −80°C prior to RT-qPCR. RT-qPCR for SARS-CoV-2 was performed using the TaqPath™ 1-Step RT-qPCR Master Mix, CG (Applied Biosystems) and the Centers for Disease Control and Prevention (CDC) N1 assay (Integrated DNA Technologies). Thermal cycling was performed with the QuantStudio™ 6 Pro (Thermo Fisher Scientific). All samples were analyzed in duplicate. Viral RNA copies were calculated from a standard curve constructed with the N gene positive control plasmid (Integrated DNA Technologies) and multiplied by the dilution factors to obtain copies in original samples. The detection cut-off was calculated from the limit of detection multiplied by the dilution factor of the samples, i.e., 40 RNA copies for air, and 90 RNA copies for surface samples.

### Viral cultures

Selected samples with low threshold cycle (Ct) values (top 3 air, top 3 surface swab, and 1 RT-qPCR negative from each sample type) were cultured in a 12-well plate of confluent A549-ACE2 cells using 25% of the original sample volume. Cultures were passaged onto a new plate at 3 days post infection. Both plates were observed daily for cytopathic effect and harvested at 6 days post infection.

### Statistical analysis

Each datapoint of our analyses represent an individual collection performed in separate locations, or on different days. Four datapoints from sampling with mask (surgical/N95) were not included in the analyses except for the mask comparison collection. Analyses and graphs were performed with GraphPad Prism version 9.0. Individual statistical analyses are denoted in the figure legends. Statistical significance was defined as having a *p*-value of *p* < 0.05.

## Results

We aimed to evaluate air sampling for mass surveillance in hospital settings that can aid detection of nosocomial pathogens as well as inform containment design for future outbreaks with potential aerosol transmission. Therefore, our study focuses on assessing the duration, consistency and distance of aerial surveillance using an active air sampling device (AerosolSense^TM^). The raw detection values of the sampling results are listed in Supplementary Table 1 (air sampling) and Supplementary Table 2 (surface sampling).

### SARS-CoV-2 RNA can be detected with short air sampling times

**Figure 2:**
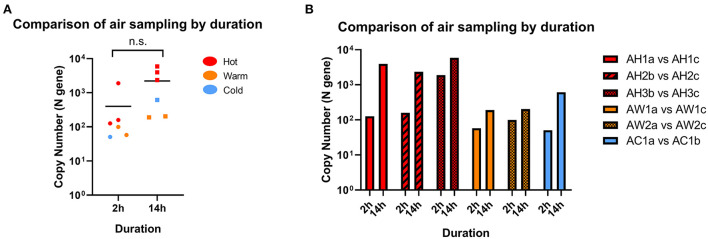
Comparison of SARS-CoV-2 detection by sampling duration. **(A)** Comparison of RNA copy number in air samples collected from the same location following 2 or 14 h of sampling. Statistical significance was calculated using a two-sample *t*-test, *n* = 6. **(B)** Paired samples from individual locations with different sampling time. *n* = 1 for each paired location.

We first assessed the duration of air sampling that can enable the detection of SARS-CoV-2 RNA by comparing sampling times of 2- and 14-h in Hot, Warm, and Cold areas. We found sufficient RNA above the detection cut-off to be considered positive for both sampling times in all locations, and the differences were not statistically significant (Figure 2A). Interestingly, we observed positive detection in Warm and Cold areas for both sampling times, which are designated “clean” areas according to hospital guidelines, albeit at lower levels compared to Hot areas. When further categorized based on C+ patient traffic, SARS-CoV-2 detection levels were mostly comparable across the sampled sites (Figure 2B). Therefore, for subsequent analyses, all data points across different sampling durations were included.

### Air sampling is a more consistent method of detection compared to surface swab in C+ facilities

**Figure 3:**
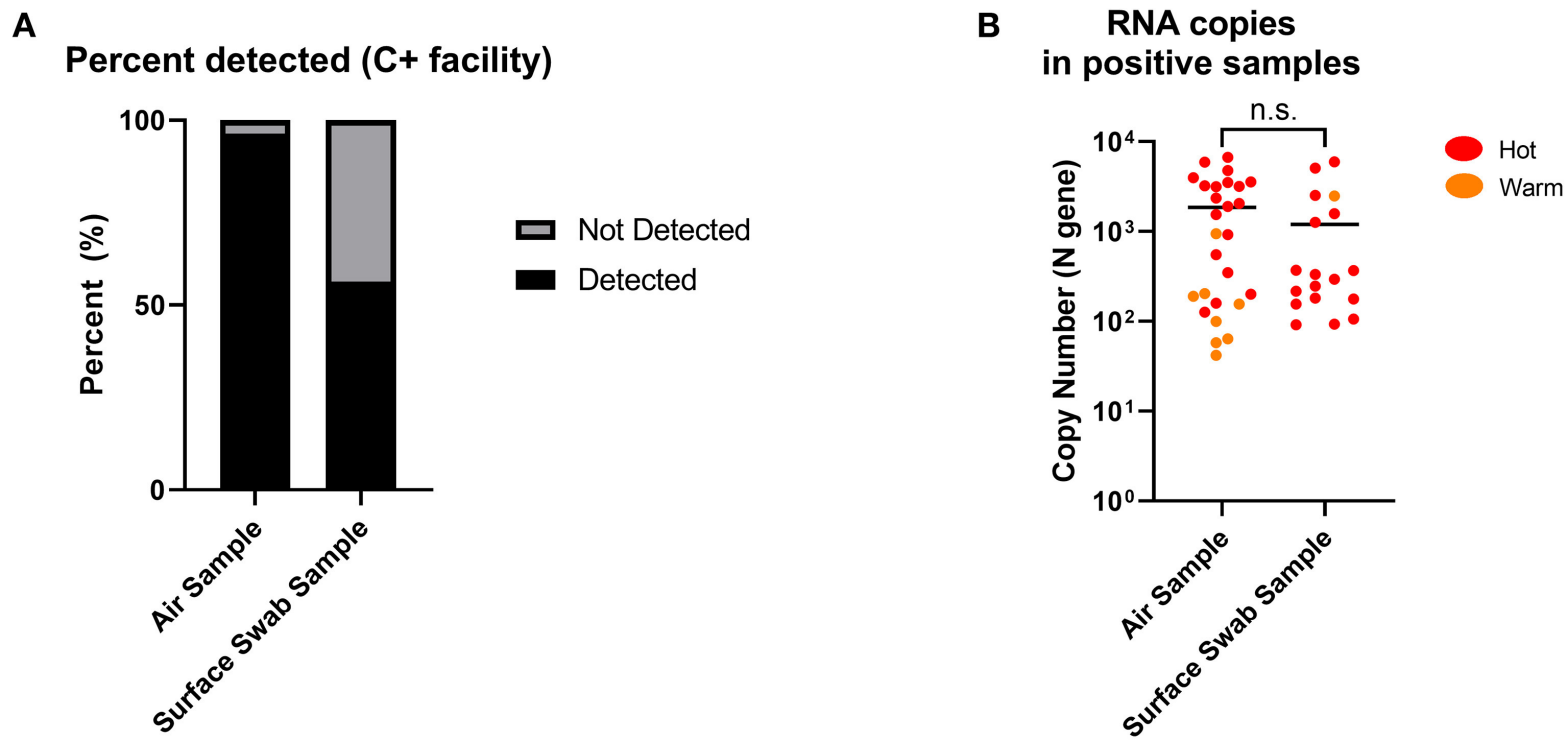
Positive detection of SARS-CoV-2 RNA copies from air and surface swab samples in C+ facilities. **(A)** Detection of SARS-CoV-2 RNA copies were found in 27 out of 28 (96.4%) air samples compared to 18 out of 32 (56.3%) surface swab samples. Positive detection was defined as being above the detection limit of the RT-qPCR performed on the collected samples, factoring in the dilution factor of the sample collection fluid. Detection limits: 40 RNA copies (air); 90 RNA copies (surface swab). **(B)** SARS-CoV-2 RNA copies in positive samples from C+ facilities (Hot and Warm areas) were comparable between air and surface swab samples. Statistical significance was calculated using a two-sample *t*-test.

Twenty-eight air samples (excluding masked samples) were collected and processed for SARS-CoV-2 RNA by RT-qPCR. Of the 28 samples (19 from Hot and 9 from Warm), 27 returned positive (96.4%), while 1 was negative. All 19 of the Hot samples were positive (100%), while 8/9 of the Warm samples were positive (88.9%). Concurrently, 32 surface swab samples (29 from Hot and 3 from Warm) were obtained from C+ facilities during the same collection window, and only 18 samples returned positive (56.3%). Among them, 17/29 of the Hot samples returned positive (58.6%) while 1/3 of the Warm samples returned positive (33.3%; Figure 3A, Supplementary Figures 3, 4). While the detection cut-off for surface sampling was higher than that of air sampling (90 vs. 40 RNA copies), almost all the negative surface detection (13/14) were a result of non-detection of SARS-CoV-2 RNA (N.D, no Ct value), as opposed to none from the air sampling in C+ facilities (Supplementary Tables 1, 2). Among the positive detections, the levels of detection between air and surface sampling were shown to be comparable with no significant differences (Figure 3B). This suggests that while both sampling methods can retrieve similar levels of SARS-CoV-2 RNA, air sampling can provide more consistent detection results compared to surface sampling, where detection is dependent on the area swabbed. In addition, it is interesting that we found positive detection in Warm areas which are designated “clean” areas. This highlights the need for a sensitive surveillance system for consistent detection of possible contamination to inform further adjustments in containment measures, or to identify pre-symptomatic individuals who were falsely negative by hospital admission ART.

### SARS-CoV-2 RNA can be detected across long distances *via* air sampling

**Figure 4:**
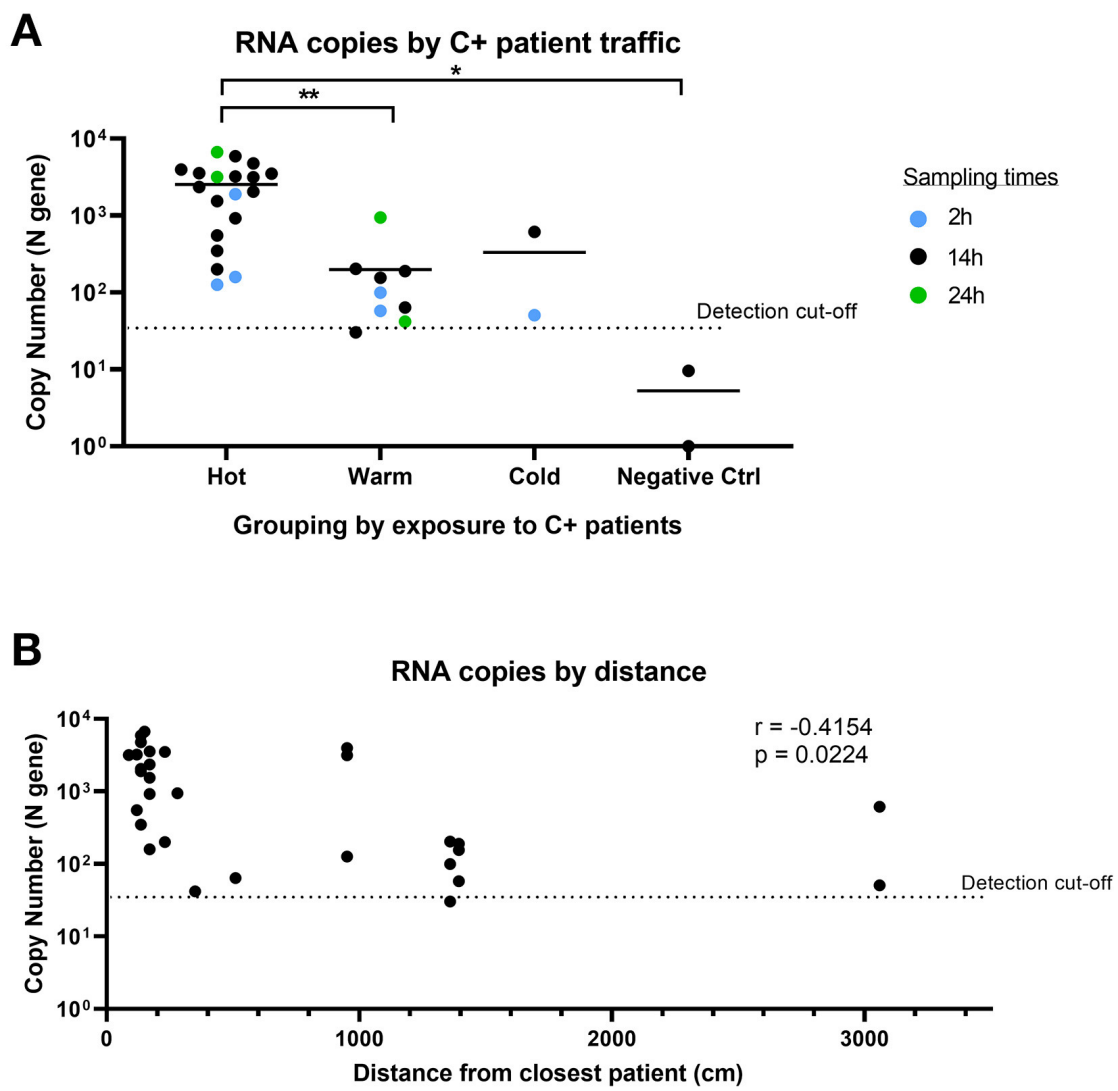
Comparison of SARS-CoV-2 detection by sampling location and distance. **(A)** SARS-CoV-2 copy number grouped by location of exposure to SARS-CoV-2. Hot: areas with C+ patient traffic; Warm: areas in COVID-19 wards with no C+ patient traffic; Cold: areas not expected to have C+ patients. *n* = 32 (excludes masked samples). Groups were compared using non-parametric Kruskal-Wallis test. **(B)** Comparison of RNA copy number by distance from closest C+ patient for air samples. *n* = 30 (excludes masked samples and negative controls). Correlation analysis was conducted using Pearson's correlation.

We observed consistent detection of SARS-CoV-2 RNA in Warm and Cold areas (“clean” areas). Despite being designated as a “clean” area with no COVID-19 patients, both of the air samples collected in Cold areas returned positive for SARS-CoV-2 detection. As negative controls, we collected air samples from an adjacent office building, which returned negative for SARS-CoV-2 detection. This suggests that the positive detection from the Cold area was not a false positive result. When we further analyzed the SARS-CoV-2 copy number detected in each area (Figure 4A), we found that there was a significant difference between the Hot and Warm areas, attributed to the presence of C+ patients in Hot areas. The level of detection was similar in Warm and Cold areas, which are both “clean” areas. We also compared the levels of SARS-CoV-2 RNA detected based on proximity to the closest bed assigned to a COVID-19 patient and whether there was physical separation from COVID-19 patients. SARS-CoV-2 RNA was found in generally lower quantities in locations further away from COVID-19 patients (*r* = −0.4154; Figure 4B). However, SARS-CoV-2 RNA could still be detected more than 30 m away from the closest COVID-19 patient. Our data suggests that despite physical designation of “contaminated” and “clean” areas by hospitals, SARS-CoV-2 can still be detected in proximate areas within the same building, during times when large numbers of infected patients are present. This suggests that the air sampling device can potentially be used to detect aerial contamination in a building to inform decisions regarding more effective containment measures.

### SARS-CoV-2 RNA can be detected by the air sampler when “masked”

**Figure 5:**
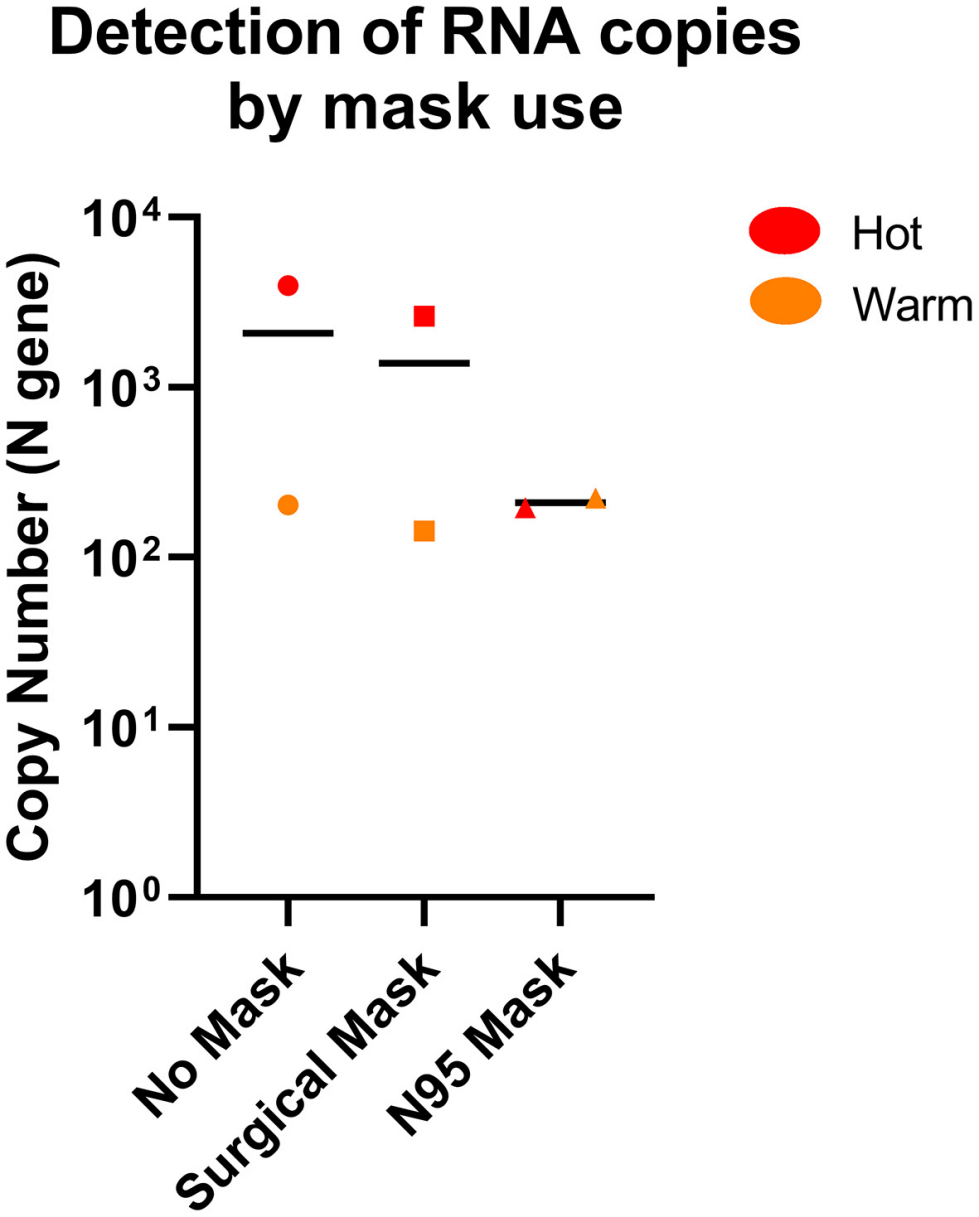
Comparison of SARS-CoV-2 detection by the AerosolSense^TM^ sampler with or without mask. SARS-CoV-2 RNA was detected in both Hot and Warm locations with or without mask. *n* = 2 per group.

We also performed a limited test of air sampling where the sampler inlet was covered by either a surgical or N95 mask, compared to a sampler without mask cover in Hot and Warm areas to simulate both “clean” and “dirty” environments (Supplementary Table 3). The air sampler was able to detect SARS-CoV-2 through masks when sampled for 14-h, albeit at a potentially lower level for a N95 mask, but not for surgical mask (Figure 5). This suggests that the strength of the sampler is sufficient to negate the mask effect when sampled for longer durations and suitable for hospital surveillance where mask usage is common.

### Samples with positive SARS-CoV-2 RNA detection were not viable when cultured

Lastly, we selected three samples each from air and surface sampling with the highest copy number (lowest Ct values) and performed viral culture of these samples. We were unable to retrieve viable virus from two passages based on the lack of cytopathic effect in all cultures. RT-qPCR was also performed on the viral culture supernatants which confirmed the lack of viral replication.

## Discussion

Over the past 2 years, different infection control measures have been implemented to contain the SARS-CoV-2 virus, with varying degrees of success (3, 29, 30). As regions shift toward endemicity, SARS-CoV-2 surveillance will be a key component of public health measures to pre-empt surges and manage healthcare resources accordingly. This is in particular due to the potential airborne transmission of SARS-CoV-2 (31–33), the emergence of the relatively more transmissible but less virulent Omicron VOC (27), a large number of asymptomatic infections (34), and pre-symptomatic transmissions (35) that have been reported since the beginning of the pandemic. Hospitals remain a high-risk location for nosocomial outbreaks (36), regardless of high or low community caseloads. While many mitigation measures worked during the pandemic (7, 21, 22), the resource intensiveness of these measures warrants further updates on handling nosocomial outbreaks and hospital transmission. In our study, we performed air sampling with the AerosolSense^TM^ to demonstrate, as a preliminary proof-of-concept, that the method was sensitive and applicable for mass surveillance of SARS-CoV-2 in the hospital setting (18, 37).

Air sampling surveillance has been developed and conducted during both the current COVID-19 pandemic and past outbreaks (19, 38, 39). Early studies have shown that SARS-CoV-2 can be found in the environment, especially in areas close to the infectious sources, carried by droplets and possibly aerosols generated by infected individuals (5, 40, 41). Expanding on previous controlled testing of the device (37), our results provided additional evidence in a healthcare setting that demonstrated a sensitive, consistent and high rate of detection of viral RNA found in almost all locations sampled, even in designated “clean” areas, and with great distances (up to 30 m) away from infected patients. Our data indicates that the AerosolSense^TM^ device had consistent detection across long distances in well-ventilated spaces. Additionally, the high flow rate of 200 L/min allows detection in short time frames. These features are a combination of the individual strengths of different sampling methods reported in previous studies and highlights the utility of the device we tested (18, 42, 43). In addition, findings of long-distance aerial dispersal have been reported (43, 44), which further affirms the utility of this device for long-range detection. Furthermore, the device we employed utilized liquid-coated collection substrates, which can improve the retrieval rate of airborne pathogens such as SARS-CoV-2 (45). Notably, we were able to detect viral RNA through N95 respirators and surgical masks in the C+ facility (Hot and Warm areas). This suggests that air sampling is suitable as a surveillance method in areas with mask usage, such as in healthcare settings which require staff to don PPE. At the same time, this also emphasizes the potential limitations of non-pharmaceutical interventions in preventing transmission and highlights the effectiveness of a surveillance system to complement public health measures (46, 47).

Air surveillance sampling offers several advantages, as shown by our results, such as localized coverage of entire rooms or floors in the built environment, near real-time results (in as little as 2 h of sampling), and sensitive detection from a pooled source. The device is portable with no installation requirements, allowing rapid deployment in hospital wards and other healthcare facilities (e.g., quarantine and community isolation centers). Employing this additional layer of aerial surveillance can help to further subvert risk of transmission and nosocomial outbreaks in hospital and healthcare settings, providing added protection to healthcare workers and high-risk patients (48–50). Furthermore, the air sampling device can be used to test new prevention and containment measures in future hospital or clinic building designs.

In comparison, environmental surface sampling (51), was found to vary more and may be dependent on the location swabbed, traffic, and cleaning frequency. This is especially apparent when surface samples tend to return higher detection levels at high touch surfaces, while low touch areas may result in false negatives. Therefore, a high number of surface samples would be required to adequately assess an area, which may not be a sustainable practice. This is circumvented by air sampling which can cover a wider area and over time, with a manageable number of units and samples. We did not perform surface sampling in the Cold areas as our previous study showed no detection in C- facilities (18). Indeed, this was further observed in the current study where surface sampling more frequently returned non-detection, even in Hot areas. Thus, the comparison between air and surface sampling in our study highlighted the higher coverage and sensitivity of air sampling for the detection of nosocomial pathogens in hospitals.

The AerosolSense^TM^ air sampling device may also be potentially employed in a pathogen agnostic manner for detecting nosocomial infections, due to its ability to detect different pathogens when coupled with molecular techniques like RT-qPCR (20). Such routine surveillance of the “air microbiome” has been proposed and can be applied to healthcare settings in nosocomial infection surveillance, although the current focus has been on fungal and bacterial organisms rather than viruses (52). With the air sampling techniques developed during the pandemic, it is now possible that routine surveillance of the “air microbiome” might allow for better design of healthcare buildings and surveillance system in preventing nosocomial infections and protecting healthcare staff. This is also congruent with the socio-behavioral changes in the population post COVID-19 pandemic, which emphasizes aerosol/airborne transmission control and healthcare worker protection to mitigate infection risks. The deployment of aerial surveillance devices could create a safe hospital environment and improve public confidence in the continual usage of healthcare facilities (53). In addition, the emergence of Omicron and its subvariants with improved transmission also indicates the need for more effective surveillance on potentially airborne pathogens as added measures to inform infection control (6, 54–56).

Our study, however, is not without its limitations. Firstly, we did not map airflow vectors in the environments sampled, which could have resulted in false negative surveillance of the air. We rectified this limitation by placing samplers within patient respiratory exhalation dispersion areas, sampled for longer periods of time, and found that we were able to detect SARS-CoV-2 in almost every area sampled. Secondly, our sample size and sampling areas were small, which may not reflect the same layouts as other healthcare facilities. Although we did try to cover both NP isolation wards and cohort wards, we could not cover all the different clinical settings. We also had small sample sizes for the C- Cold areas and mask study. Hence, the findings on sampling distance and mask usage are preliminary and warrant further studies. Thirdly, while we detected SARS-CoV-2 RNA, viable virus was not retrieved from cultures. This may be due to several reasons, such as shedding of non-viable virus, levels of viable virus below what can be detected, or collection technique impacting viral viability. Sampling techniques from the environment often entails harsh mechanical collection that may impact even direct collections from infected patients (5). Therefore, high flow rate sampling devices, such as the AerosolSense^TM^ used in this study, may not be suitable for transmission studies that require detection of live viruses. Nevertheless, it remains a powerful qualitative tool for surveilling environmental pathogens in hospitals to detect the potential for nosocomial outbreaks before they occur, protecting healthcare workers and vulnerable, high-risk patients. Lastly, due to the limited scope of the study, we did not have sufficient data to evaluate the airborne transmission risks. However, we reported standardized quantified viral loads, allowing future studies to utilize our results for transmission modeling.

In conclusion, for viruses and pathogens with airborne transmission potential, a sensitive air sampling method will allow mass surveillance and monitoring of viral presence in healthcare settings with increased transmission risk. Our study provided proof-of-concept for future studies to optimize protocols for such surveillance that can detect and prevent nosocomial outbreaks early. The surveillance strategy employed in this study can be rapidly adapted due to its pathogen agnostic nature. In addition, the same sampling equipment can be used for the detection of novel pathogens when paired with molecular technologies (multiplex qPCR or unbiased next generation sequencing), improving our capabilities in screening for the likely disease X candidates during their early emergence when patients present themselves. The utility of such a device and sampling method may be vital for future infection control, especially with globalization encouraging the transmission of respiratory infectious diseases.

## Author's note

Sequence of first authors with equal contributions was decided by drawing lots.

## Data availability statement

The original contributions presented in the study are included in the article/Supplementary material, further inquiries can be directed to the corresponding authors.

## Ethics statement

As there was no direct use of patient samples or identifiers involved, the study was given a waiver of approval by the Domain Specific Review Board (DSRB) of the National Healthcare Group (NHG).

## Author contributions

KT, AA, DT, JS, JC, PT, and DA: concept and design of project. KT, AA, DT, LP, and AN: acquisition of data. KT, AA, DT, JS, PT, and DA: analysis and interpretation of data. JC, PT, and DA: funding acquisition. KT, AA, DT, JS, AN, PT, and DA: drafting and revising of manuscript. All authors have read through and agreed on the final version of the manuscript draft prior to submission.
