# Supplementary material for: Detection of hospital environmental contamination during SARS-CoV-2 Omicron predominance using a highly sensitive air sampling device
Source: Front Public Health. 2023 Jan 10;10:1067575. doi: 10.3389/fpubh.2022.1067575 (PMC9873263; doi:10.3389/fpubh.2022.1067575)
Supplement: Supplementary file 1 [file Data_Sheet_1.docx]

**Supplementary Materials**


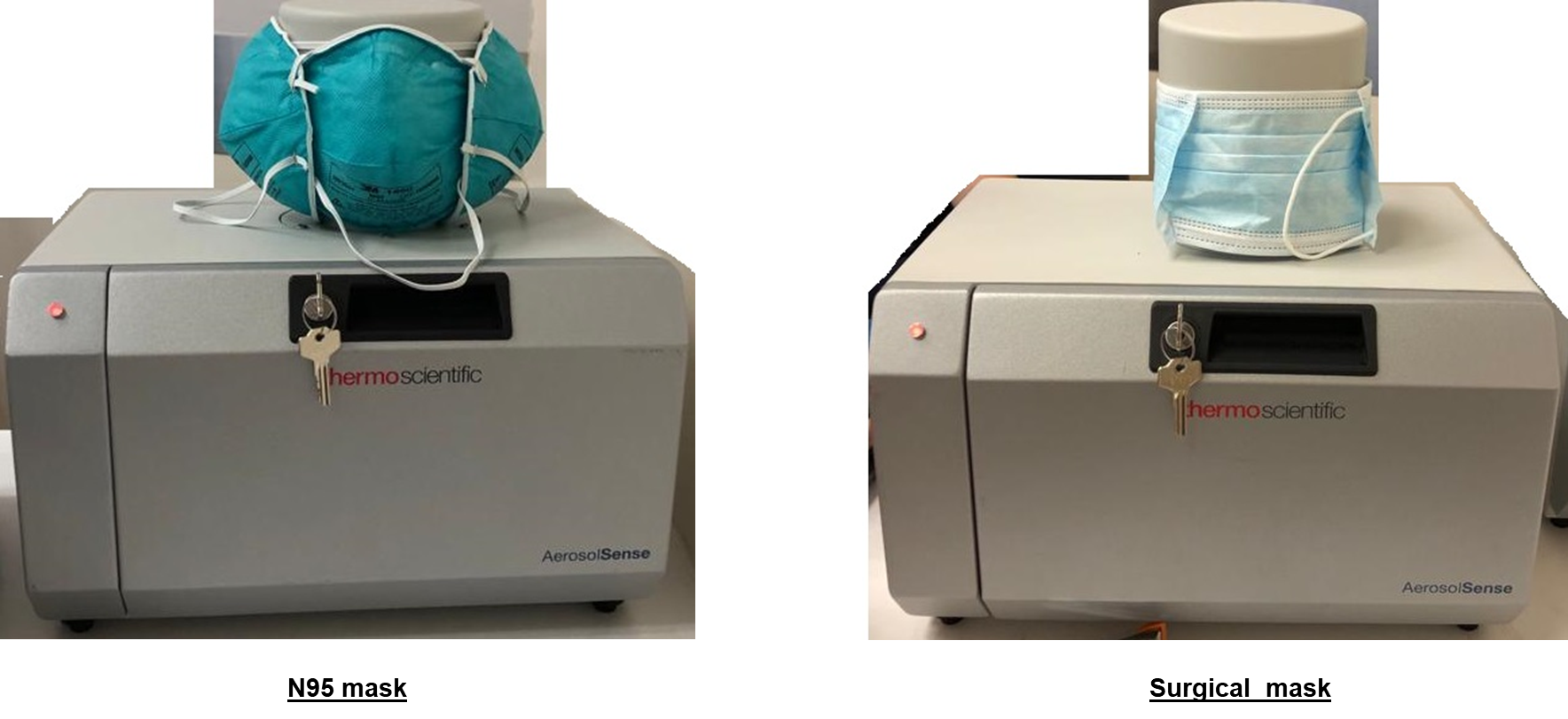


**Figure S1.** Image of mask fitting (N95 and surgical) over the AerosolSense™ air sampler’s inlet.


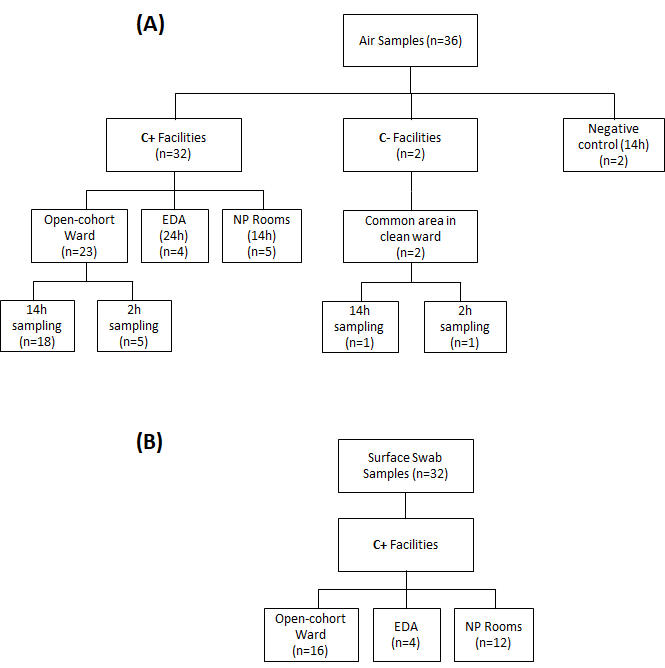


**Figure S2.** Flowchart of (A) air and (B) surface swab sampling workflow in the hospital setting.


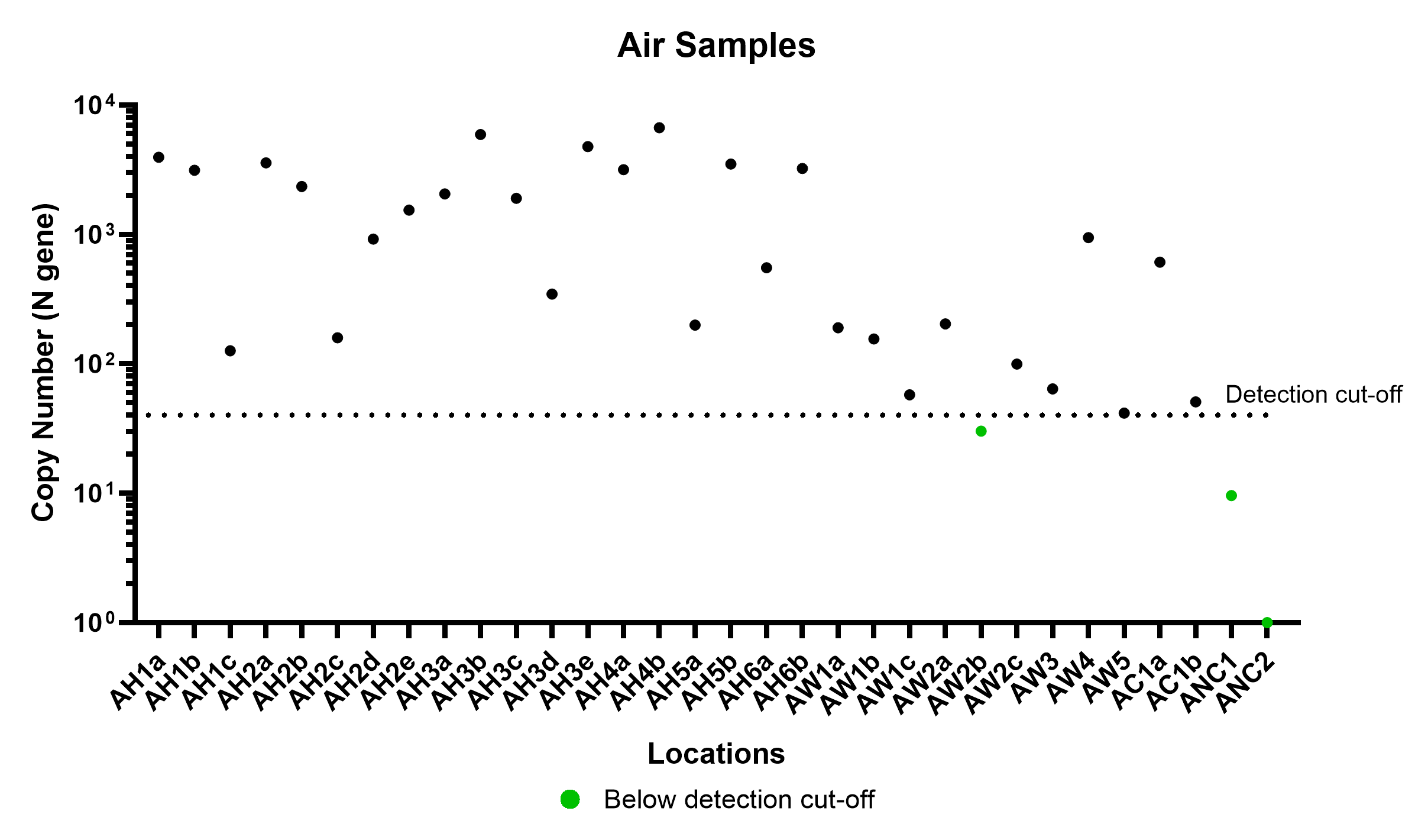


**Figure S3.** Copy number of air samples by location. A value of 1 was given to samples not detected by RT-qPCR to appear on the log_10_ scale. n=32 (excludes masked samples).


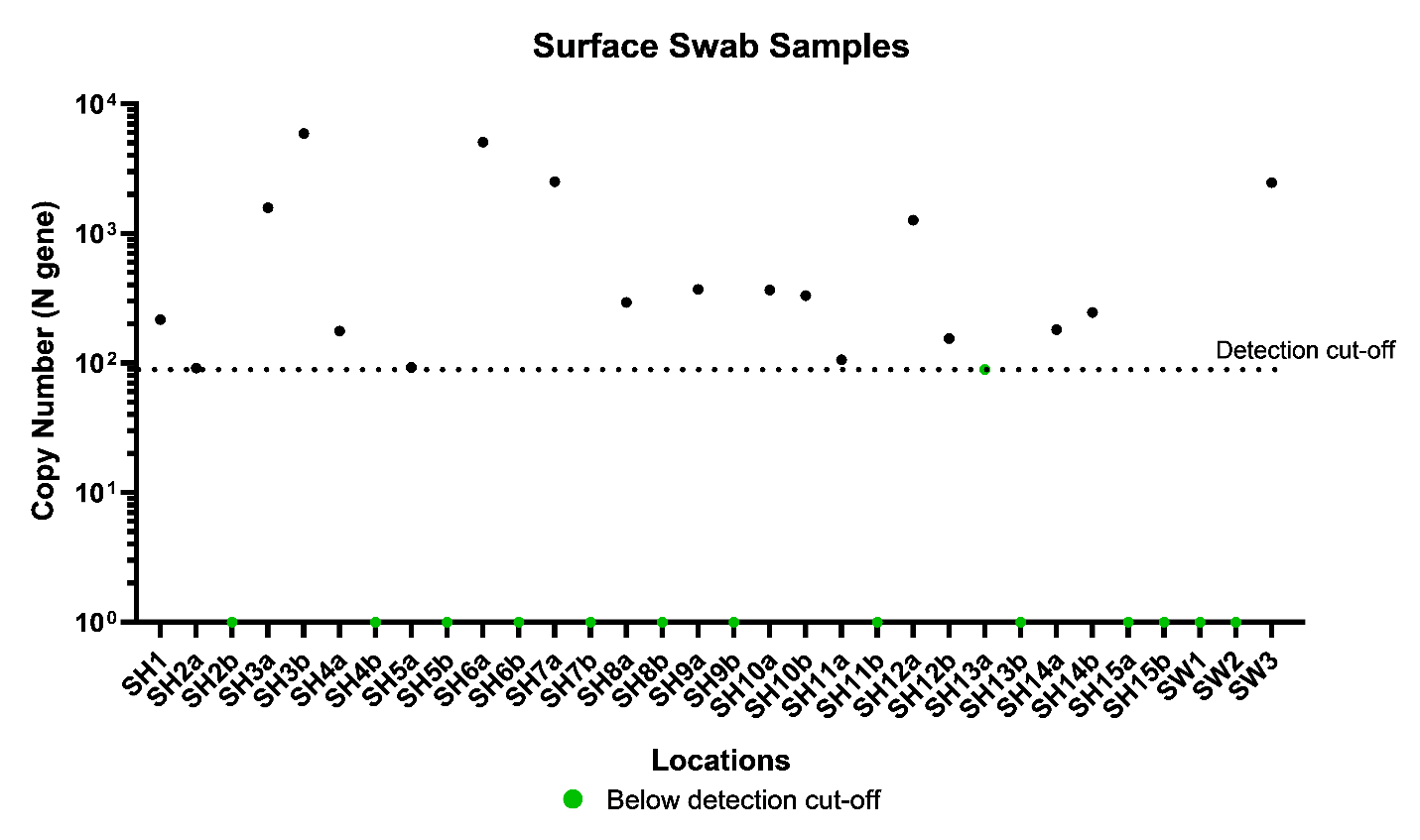


**Figure S4.** Copy number of surface swab samples by location. A value of 1 was given to samples not detected by RT-qPCR to appear on the log_10_ scale. n=32.

**Supplementary Tables**

**Table S1.** Details of air sampling locations and raw data.

| Sample ID | Sample Description | Time sampled (hours) | Distance from closest patient [cm] | C+ Patient Traffic | Mean Ct | Copy number (in sample) |
| --- | --- | --- | --- | --- | --- | --- |
| AH1a | Common area in cohort ward | 14 | 952.00 | Hot | 32.43 | 3960.2 |
| AH1b | Common area in cohort ward | 14 | 952.00 | Hot | 32.76 | 3145.2 |
| AH1c | Common area in cohort ward | 2 | 952.00 | Hot | 37.34 | 126.1 |
| AH2a | Nursing area inside cohort ward cubicle 1 | 14 | 170.00 | Hot | 32.57 | 3569.8 |
| AH2b | Nursing area inside cohort ward cubicle 1 | 14 | 170.00 | Hot | 33.17 | 2347.8 |
| AH2c | Nursing area inside cohort ward cubicle 1 | 2 | 170.00 | Hot | 37.01 | 159.2 |
| AH2d | Nursing area inside cohort ward cubicle 2 | 14 | 170.00 | Hot | 34.51 | 920.7 |
| AH2e | Nursing area inside cohort ward cubicle 3 | 14 | 170.00 | Hot | 33.77 | 1546.3 |
| AH3a | Sink inside cohort ward cubicle 1 | 14 | 136.00 | Hot | 33.36 | 2055.0 |
| AH3b | Sink inside cohort ward cubicle 1 | 14 | 136.00 | Hot | 31.85 | 5916.5 |
| AH3c | Sink inside cohort ward cubicle 1 | 2 | 136.00 | Hot | 33.47 | 1905.2 |
| AH3d | Sink inside cohort ward cubicle 2 | 14 | 136.00 | Hot | 35.90 | 347.1 |
| AH3e | Sink inside cohort ward cubicle 3 | 14 | 136.00 | Hot | 32.16 | 4780.4 |
| AH4a | Patient room 1 inside emergency annex | 24 | 88.00 | Hot | 32.74 | 3176.2 |
| AH4b | Patient room 2 inside emergency annex | 24 | 150.00 | Hot | 31.68 | 6700.1 |
| AH5a | Negative pressure room 1 window | 14 | 230.00 | Hot | 36.69 | 199.7 |
| AH5b | Negative pressure room 2 window | 14 | 230.00 | Hot | 32.60 | 3510.5 |
| AH6a | Negative pressure room 1 exhaust | 14 | 119.00 | Hot | 35.23 | 553.4 |
| AH6b | Negative pressure room 2 exhaust | 14 | 119.00 | Hot | 32.72 | 3233.4 |
| AW1a | Donning area outside cohort ward | 14 | 1394.00 | Warm | 36.75 | 190.4 |
| AW1b | Donning area outside cohort ward | 14 | 1394.00 | Warm | 37.04 | 155.9 |
| AW1c | Donning area outside cohort ward | 2 | 1394.00 | Warm | 38.45 | 57.8 |
| AW2a | Staff area outside cohort ward | 14 | 1360.00 | Warm | 36.66 | 204.0 |
| AW2b | Staff area outside cohort ward | 14 | 1360.00 | Warm | 39.38 | 30.3 |
| AW2c | Staff area outside cohort ward | 2 | 1360.00 | Warm | 37.68 | 99.8 |
| AW3 | Negative pressure room 1 anteroom | 14 | 510.00 | Warm | 38.31 | 64.0 |
| AW4 | Staff area inside emergency annex | 24 | 280.00 | Warm | 34.47 | 947.4 |
| AW5 | Pantry inside emergency annex | 24 | 350.00 | Warm | 38.92 | 41.7 |
| AC1a | Common area in clean ward | 14 | 3060.00 | Cold | 35.09 | 612.1 |
| AC1b | Common area in clean ward | 2 | 3060.00 | Cold | 38.64 | 50.8 |
| ANC1 | Empty room | 14 | Negative control | NC | 41.02 | 9.6 |
| ANC2 | Storage room | 14 | Negative control | NC | N.D | N.D |

**Table S2.** Details of surface sampling locations and raw data.

| Sample ID | Sample Description | C+ Patient Traffic | Mean Ct | Copy number (in sample) |
| --- | --- | --- | --- | --- |
| SH1 | Counter of common area in cohort ward | Hot | 37.73 | 216.7 |
| SH2a | Nursing counter inside cohort ward cubicle 1 | Hot | 38.95 | 91.9 |
| SH2b | Nursing counter inside cohort ward cubicle 2 | Hot | N.D | N.D |
| SH3a | Sink inside cohort ward cubicle 1 | Hot | 34.89 | 1584.8 |
| SH3b | Sink inside cohort ward cubicle 2 | Hot | 33.01 | 5939.9 |
| SH4a | Cardiac table inside cohort ward cubicle 1 | Hot | 38.02 | 176.9 |
| SH4b | Cardiac table inside cohort ward cubicle 2 | Hot | N.D | N.D |
| SH5a | Nurse call button inside cohort ward cubicle 1 | Hot | 38.93 | 92.9 |
| SH5b | Nurse call button inside cohort ward cubicle 2 | Hot | N.D | N.D |
| SH6a | Toilet sink inside cohort ward cubicle 1 | Hot | 33.23 | 5085.4 |
| SH6b | Toilet sink inside cohort ward cubicle 2 | Hot | N.D | N.D |
| SH7a | Toilet bowl inside cohort ward cubicle 1 | Hot | 34.23 | 2513.3 |
| SH7b | Toilet bowl inside cohort ward cubicle 2 | Hot | N.D | N.D |
| SH8a | Toilet door handle inside cohort ward cubicle 1 | Hot | 37.29 | 294.1 |
| SH8b | Toilet door handle inside cohort ward cubicle 2 | Hot | N.D | N.D |
| SH9a | Toilet door handle inside negative pressure room 1 | Hot | 36.96 | 371.0 |
| SH9b | Toilet door handle inside negative pressure room 2 | Hot | N.D | N.D |
| SH10a | Nurse call button inside negative pressure room 1 | Hot | 36.97 | 367.3 |
| SH10b | Nurse call button inside negative pressure room 2 | Hot | 37.12 | 332.4 |
| SH11a | Sink inside negative pressure room 1 | Hot | 38.74 | 106.3 |
| SH11b | Sink inside negative pressure room 2 | Hot | N.D | N.D |
| SH12a | Toilet sink inside negative pressure room 1 | Hot | 35.21 | 1267.2 |
| SH12b | Toilet sink inside negative pressure room 2 | Hot | 38.20 | 155.5 |
| SH13a | Toilet bowl inside negative pressure room 1 | Hot | 38.99 | 89.1 |
| SH13b | Toilet bowl inside negative pressure room 2 | Hot | N.D | N.D |
| SH14a | Cardiac table inside negative pressure room 1 | Hot | 37.98 | 181.9 |
| SH14b | Cardiac table inside negative pressure room 2 | Hot | 37.55 | 245.9 |
| SH15a | Cardiac table inside patient room 1 inside emergency annex | Hot | N.D | N.D |
| SH15b | Cardiac table inside patient room 2 inside emergency annex | Hot | N.D | N.D |
| SW1 | Keyboard in staff area outside cohort ward | Warm | N.D | N.D |
| SW2 | Keyboard in staff area inside emergency annex | Warm | N.D | N.D |
| SW3 | Table in pantry inside emergency annex | Warm | 34.25 | 2482.6 |

**Table S3.** Details of air sampling locations and raw data for mask collection study.

| Air sample for mask comparison | | | | | | |
| --- | --- | --- | --- | --- | --- | --- |
| Sample ID | **Sample Description** | **Time sampled (hours)** | **Distance from closest patient [cm]** | **C+ Patient Traffic** | **Mean Ct** | **Copy number (in sample)** |
| AH1a | Common area in cohort ward - Original sample control | 14 | 952.00 | Hot | 32.43 | 3960.2 |
| AH1d | Common area in cohort ward - Surgical mask | 14 | 952.00 | Hot | 33.01 | 2623.2 |
| AH1e | Common area in cohort ward - N95 mask | 14 | 952.00 | Hot | 36.72 | 195.7 |
| AW2a | Staff area outside cohort ward - Original sample control | 14 | 1360.00 | Warm | 36.66 | 204.0 |
| AW2d | Staff area outside cohort ward - Surgical Mask | 14 | 1360.00 | Warm | 37.16 | 143.4 |
| AW2e | Staff area outside cohort ward - N95 Mask | 14 | 1360.00 | Warm | 36.53 | 222.6 |
